# ‘At the right time, in the right way, with the right resources’: perceptions of the quality of care provided during childbirth in Malawi

**DOI:** 10.1186/1471-2393-14-248

**Published:** 2014-07-28

**Authors:** Elizabeth O’Donnell, Bettina Utz, Diana Khonje, Nynke van den Broek

**Affiliations:** Liverpool School of Tropical Medicine, Pembroke Place, Liverpool, L3 5QA UK; Reproductive Health Unit, Ministry of Health, Lilongwe, Malawi

**Keywords:** Quality of care, Maternity care, Autonomy, Resource poor setting

## Abstract

**Background:**

Improving the quality of care women receive during childbirth is as important as ensuring increased availability of care and numbers of healthcare providers. To be able to improve quality of care, it is important to understand what quality means for mothers as well as providers of care.

**Methods:**

33 postnatal mothers and 10 healthcare providers from all 4 major hospitals in one district in Malawi were interviewed via 27 in-depth interviews and 2 focus group discussions. Data was transcribed and analysed using the thematic framework approach.

**Results:**

Perceptions of quality of care differed substantially between care providers and postnatal mothers. For caregivers, characteristics of good quality care included availability of resources while for postnatal mothers positive relationships with their caregiver were important. Lack of autonomy and decision making power is a barrier to quality of care and it exists both at the level of the patient (mother) and at the level of her caregiver with healthcare providers unable to influence decisions made by more senior staff or management. Lack of autonomy was linked with the emerging themes of staff de-motivation, frustration, lack of empowerment to make change and resulting in a poor quality of care provided.

**Conclusions:**

Creating a reciprocal understanding of what good quality care comprises and the barriers as well as promoters of this should be the starting point for improving the quality of maternity care. A renewed focus is needed on improving communication, strengthening patient rights and autonomy whilst simultaneously motivating and enabling healthcare workers to provide comprehensive and inclusive quality of care.

## Background

The proportion of births attended by a skilled birth attendant (SBA) defined as a provider who is “trained to proficiency in the skills needed to manage normal (uncomplicated) pregnancies, childbirth and the immediate postnatal period, and in the identification, management and referral of complications in women and newborns” [1] is an important indicator for monitoring progress toward Millennium Development Goal (MDG) 5. The international target to have 90% of births attended by skilled health personnel by 2015 remains a major challenge [1].

Worldwide, many efforts have been made to address this challenge and in the past decade a major focus has been placed on improving access to skilled birth attendance and Emergency Obstetric Care. Particularly in countries with poor infrastructure more hospitals have been built, more skilled birth attendants have been trained, and through work education and community initiatives more families have been encouraged to give birth in health facilities.

By 2008, 66% of all births globally were attended by a SBA. By 2012, the proportion is still low in the African region (49%) compared to 59%, 94% and 92% in Asia, the Americas and Europe respectively [2, 3]. The global focus on increasing uptake of and demand for facility based care to reduce maternal mortality creates an urgent need to address the quality of maternal care [4].

There is emerging evidence that when the quality of care at a health facility level is perceived to be poor, this discourages women and their families from accessing this care. Similarly, a poor quality of care or care that is ‘sub-standard’ is recognised to result in increased morbidity and mortality. To improve the quality of maternity care an understanding of what is meant by the concept of “quality of care” is important. A systematic literature review showed that there is no universally accepted definition of quality of care and the multi-faceted nature of quality is widely acknowledged. Models describe quality of care from the perspective of health care providers, managers and patients; dimensions within the health care system; using elements such as safety, effectiveness, patient-centeredness, timeliness, equity and efficiency; and through the provision of care and experience of care [5].

Although several definitions and frameworks have been developed to define quality of care, defining the quality of care in maternal and neonatal health remains a challenge due to a multitude of aspects that have to be considered. Hulton et al have highlighted the fact that in the context of maternal health and child birth, effective (safe) care, timely access and reproductive health rights are all important components of the quality of care provided [6].

Both the quality of the provision of care by the healthcare provider (birth attendant) and the quality of care as experienced by users are important. The use or uptake of services, including care at the time of childbirth is the result not only of the availability of that care but also of women’s experiences of that care [7]. Maternity audit, in a variety of forms, is now being implemented in many resource-poor countries. All essentially ask the same three questions: what was done well, what was not done well, and how can care be improved in future? [8, 9] However, provision of care may be deemed of high quality against recognised clinical standards of care but unacceptable to the woman and her family. Conversely, some aspects of care may be popular with women but may be ineffective or harmful to health.

This study aimed to explore the perceptions of maternity care from the point of view of both the mother who had received maternity care and the healthcare provider who had provided care in a rural setting in Malawi.

## Methods

The study was conducted in the four hospitals of Mangochi district (Mangochi District General, St. Martins, Monkey Bay and Mulibwanji). The facilities were purposefully selected to guarantee a sufficient number of daily deliveries but also ensure a representative number of study participants. All study hospitals had quality improvement committees and conducted maternal deaths audits.

The study population included postnatal mothers aged between 16 to 36 years who had delivered in the last 7 days. We excluded mothers with poor birth outcomes such as stillbirths or neonatal deaths from focus group discussions as we considered it unethical and insensitive to group them with healthy mothers and healthy babies.

The healthcare providers included in this study were all working on the maternity wards. Data was collected by the main researcher and four trained national research assistants who understood the local language using in-depth semi-structured interviews (IDI) and focus group discussions (FGD). In order to guarantee the consistency of the interviews, all research assistants received training in qualitative data collection and used the same topic guides. Topic guides addressed the understanding of the term “quality of care”, factors contributing to good or poor quality of care and the interviewees’ perceptions and experiences of the quality of care provided or received during childbirth. 33 postnatal mothers and 10 health care providers participated in the study; a total of 16 mothers attended the two FGD, 17 mothers and 10 healthcare providers (4 midwives, 4 clinical officers and one nurse attendant) participated in IDI. All participants were chosen purposefully to ensure a varied sample of postpartum mothers and healthcare providers with different levels of experience and care roles (midwives, nurses, clinical officers).

Data was recorded, translated into English and transcribed. All data was analysed using the framework approach [10] and NVivo software version 8.0.

### Ethics

All participants were informed about the purpose of the study and only participated after providing informed consent. Participants were free to withdraw from the study at any time. Ethical approval for the study was granted by the Liverpool School of Tropical Medicine Ethics Committee and the National Health Science Research Committee, Malawi.

## Results

Both women and their caregivers were eager and willing to share their understanding of and experiences with quality of care.

### Defining “Quality of Care”

Individual interpretations of the term “Quality of Care” were sought in each interview. Gaining an understanding of each participant’s interpretation of this term was important for getting an insight to their beliefs regarding care and the factors of care most important to them. The definitions of quality of care given by mothers differed from those of caregivers. For mothers, quality was related more to personal requirements: *“it is when I have soap and clean my clothes”*. In comparison, the healthcare staff defined quality in relation to their practice: “*Quality of care to me…it means giving care in terms of nursing or clinical services to the pregnant woman, or a woman who has just delivered; in the right way, at the right time, with the right resources, to the right woman”.*

### Perceptions of good quality care

**Figure 1:**
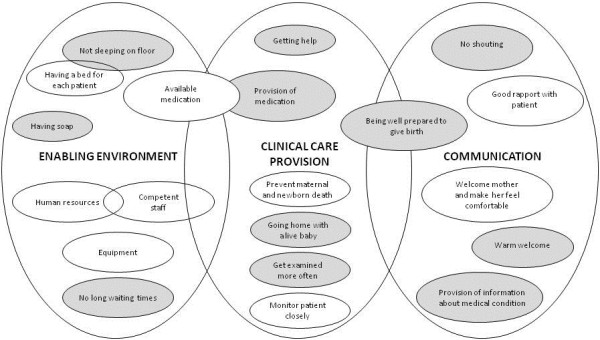
**Factors identified by both caregivers (white circles) and mothers (grey circles) as components of good quality of care.**

When asked which aspects of care were perceived to be the most important to the participants, caregivers and mothers prioritised different factors. Figure 1 summarises key factors which are arranged as representing the enabling environment, clinical care provision and as related to communication. All the caregivers listed availability of resources, such as medication, equipment and staff as the most essential components of good quality care: “*Factors that affect quality of care could be how skilled the nurse is; the availability of instruments; the availability of essential drugs”.*

The vast majority of mothers however, placed emphasis on the importance of a positive staff-patient relationship. This relationship was built by the mothers feeling welcomed into the labour environment and attended to in a timely manner as well as being able to ask questions, which was important to women. Being “shouted at” or being “scared” were considered to be poor quality care: *“[Good quality care is] when you have been received well by the staff at the hospital, and they have helped you quickly”.* It is worth noting that very few mentioned the importance of sufficient equipment and medications. In contrast, only one caregiver mentioned the importance of staff-patient relationships. Only when asked directly whether a good relationship was important, did the healthcare providers add this factor to their list of determinants. However, they also stated that ensuring a good relationship was not always feasible: *“Yes, good rapport is also important, but it is not always possible”.*

### Perceptions of barriers to good quality care

#### Lack of autonomy

Throughout the interviews participants identified lack of autonomy in the way care was given as a key barrier. This applied both to mothers making decisions regarding their own care, and to caregivers who felt they were never involved in policymaking regarding strategies to improve care. The mothers frequently commented that they did not understand the reasons why they had been given certain treatment, and their consent had often not been sought. An example of this poor communication between healthcare providers and mothers was reported by a mother who had an emergency hysterectomy. She was not informed about the details of the procedure nor that she would not be able to conceive again. *“I was bleeding so they told me that they would operate…they did not tell me what the operation was for. I found out that my baby was dead after…they did not tell me, they told my mother. I don’t know what happened, I want another baby…I do not know if this will happen”.*

#### Strained relationships and poor communication between mothers and caregivers

All the mothers interviewed expressed respect for the healthcare workers on the maternity ward. Midwives in particular were held in high regard, and their knowledge and experience gave mothers confidence. In terms of educational achievement, the healthcare providers at the four study hospitals could often speak fluent English and were relatively well educated compared to the mothers, many of whom were illiterate. However, coupled with this respect, there was a sense of fear and a belief that if a patient disagreed with, or angered a midwife, this could result in poor labour outcomes. This was illustrated by one mother who attributed the death of her sister to failure to comply to midwives advice: *“My sister was four months pregnant. She was not feeling ok and came here [the hospital] for help. She didn’t listen to what the nurses were telling her to do and so they didn’t help her. She died with the baby inside her.”*

Shouting by both mothers and caregivers on the labour ward was stated as an important factor for a strained relationship between the two. Patients admitted to screaming due to labour pain, but did not like caregivers to shout back at them. One participant spoke of a healthcare provider, all mothers hoped to avoid. “*…we have heard [from other mothers] that she shouts a lot. This is not good…when you are in pain and somebody shouts at you, you feel like its cruelty.”* Another mother gave an example of poor staff attitude and inadequate communication *“You are in crying pain, but sometimes a nurse doesn’t come to help because she is busy on the mobile [phone]…if she does come she shouts at you for screaming”.* Interviewed mothers considered this behaviour as wrong: *“It is not good for patients to be shouted at”.* They reflected that such experiences could negatively impact future health seeking behaviour of pregnant mothers.

Caregivers reported that the pressure of their jobs could result in “being stressed” and in “a bad attitude” towards patients. “*Sometimes you might not answer her questions and shout at her to be quiet. Maybe I have acted that way…you are busy and frustrated and the resources are not there”.*

#### Lack of decision making power

When healthcare providers were discussing the types of poor care practices observed on their maternity wards, none of them was able to identify an official route to report negative incidents to higher authorities. This was demoralizing for staff who were eager to ensure best care for patients but felt that other team members were being negligent. *“I feel [one colleague] is incompetent…his school said he was ‘un-trainable’…he often wants to wait…we end up having to delay. One woman…he was not comfortable taking her to theatre so we had to refer her. I was told she passed away, I was furious. [If I reported him] I feel, due to low staff, they would just move him to another hospital”.* Feeling unable to formally report “incompetent” colleagues resulted in a feeling of powerlessness amongst the healthcare providers.

Midwives perceived their “lack of authority” as demoralising. When discussing midwifery training in Malawi, it emerged that many midwives felt they had a more thorough maternity training than those supervising them such as clinical officers or who, however, had the ultimate decision making powers in emergency cases. Not only did midwives feel undermined by this, but some midwives felt that, at times, patients suffered poor outcomes as a result of senior staff not agreeing with a midwife’s management plan. *“Sometimes you call for a clinician…they have not been with the mother…and your opinions [on management] collide. The midwife has spent more time in school, but the clinician has the final say…there is nothing you can do. It is frustrating as you only want the mother to benefit.”*

Likewise, healthcare providers reported a lack of feeling involved in management or policy decisions taken by hospital managers and policy makers. All caregivers who participated in the interviews enquired how the results of this study would be disseminated. They were keen to understand the views of the mothers and learn how they could improve the quality of their care giving. Many were keen to be more involved in decision making processes in the health system. The majority felt that decisions were often made without consulting them, and therefore they also assumed that effective solutions were never put into practice. *“…we have NGOs who come and undertake studies into maternity care…the results are given to higher authorities. The people high up are busy people and so results and ideas to improve do not reach us on the labour ward. We don’t get invited to presentations or meetings, but it is us who are supposed to bring about the changes. In this way I think the research may be wasted and little done”.*

## Discussion

This study revealed that there were key differences in the perception of quality of care between mothers and caregivers. While mothers prioritised the importance of a good relationship with the caregiver, healthcare providers felt the supply of resources to be most important. We also noted a lack of awareness amongst mothers regarding what they could expect with regard to “Quality of Care”. This may be due to their lower levels of education when compared to the relatively higher educational levels of healthcare providers. These findings correlate with studies suggesting that education could be a contributing factor to the differences of opinion between mothers and caregivers [11–13]. Addressing the inequalities between mothers and caregivers by strengthening education could ensure common views on quality. It may also help to improve staff-patient relationships, a key component for improved quality care [11].

A poor client-provider relationship led to feelings of powerlessness among mothers with regard to decision making about the care they required or expected during labour and delivery. This lack of autonomy has also been highlighted in a study from Ghana [14]. Health care workers mentioned the existence of a “class” hierarchy in healthcare settings and attributed the lack of autonomy to the fact that healthcare providers ‘knew what was best’. Similar findings were reported in South Africa, where prohibiting mothers from making their own health decisions was a way for caregivers to assert a higher class authority [13].

Having autonomy is not merely an ethical principle, it is a human right. To be able to take responsible decisions about their sexual and reproductive health, individuals need to be provided with adequate information resulting in informed choice which is a key condition for quality in care [15]. In our setting, healthcare workers dominated the decision making process and patients were rarely asked for their opinion. However, a more rights based approach that involves patients and builds their capacity to take decisions would be good practice as studies have shown that giving patients decision making powers is linked to improved outcomes and satisfaction [16]. Furthermore, just as dissatisfaction is shown to have implications on whether mothers access in future, improving empowerment of patients may lead to an uptake of services [11].

Poor behaviour of healthcare providers, such as shouting at mothers, was cited as an example of poor quality care. Such experiences have been described in other studies [17–20]. A lack of identified routes but also fear to report dangerous or unethical practice underlines the importance of introducing clinical audit as an effective and cheap low-technology intervention to improve quality of care [21–24]. Encouraging hospitals to harbour non-blame environments so that staff is encouraged to reflect on clinical practice, seek appraisal and perform continual professional development may change practice and improve outcomes [14].

From the point of view of the healthcare provider, many spoke of their frustration with the inability to provide what they would consider a good quality of care because of resource constraints and lack of an enabling environment. This problem has also been mentioned in an earlier publication from Malawi [25]. Such frustrations contribute to the demotivation of the healthcare provider and are likely to have a negative impact on the provision of care. Some frustrations stemmed from differences in practice and opinions between the midwives and the more senior clinicians who were said to have the ultimate decision making power.

The fact that many of the management decisions within the hospitals are made without consultation with those who are the main care providers may explain why, for example, implementation of new policy and practice fails. Not only do proposed changes often fail to reflect the local context, they can even evoke strong adverse reactions from workers [26]. It must be stressed to future policymakers that it is crucial to consider local context and seek caregivers’ opinions before changes are imposed, if effective care is to be achieved [27].

There was a feeling that the cause of poor behaviour was ultimately linked to job demotivation. Lack of motivation was influenced by the frustration and daily struggle to provide adequate care in a poorly resourced setting; a growing feeling of non-involvement and lack of teamwork or multidisciplinary approach; and a missing engagement of policy makers with caregivers on the ground. In a non-direct culture, by blaming external factors such as a lack of resources as the reason for poor quality care, disenfranchised caregivers can externalise blame and responsibility [28].

## Conclusion

By using a qualitative approach this study reflects the views of both healthcare providers and patients and provides us with identified components of care that are considered ‘good quality’. Although there are clear advantages of using such an approach this study does have several limitations. We did not include perceptions of policy makers and based on the purely qualitative approach some quantitative information such as measuring work pressure and identifying resource constraints would have added to a more comprehensive picture and would require further research. Additional research could include assessing perceptions of quality of care in the wider community and the role of participatory women’s groups and community outreach services regarding awareness raising on quality of care and its impact on the provision of quality care in the facilities.

This study highlights the need to create a reciprocal understanding of what quality comprises and provides information on factors to be addressed in order to strengthen the quality of maternity care. More focus needs to be placed on better communication between clients and providers. Such requires strengthening of patient rights and improving their autonomy whilst simultaneously supporting and motivating good practice through audits and the provision of opportunities for professional development.

## Abbreviations

MDG: Millennium Development Goal
IDI: In-depth semi-structured interviews
FGD: Focus group discussions
SBA: Skilled birth attendant.
